# Improvement in Aortic Stiffness After 9 Months of Antiretroviral Therapy in Treatment-Naïve People Living with HIV †

**DOI:** 10.3390/diagnostics16172868

**Published:** 2026-09-07

**Authors:** Pieter-Paul S. Robbertse, Anton F. Doubell, Christelle Ackermann, Innocent Maposa, Philip G. Herbst

**Affiliations:** 1 Division of Radiodiagnosis, Department of Medical Imaging and Clinical Oncology, Faculty of Medicine and Health Sciences, Stellenbosch University and Tygerberg Hospital, Cape Town 8000, South Africa; ca@sun.ac.za; 2 Division of Cardiology, Department of Medicine, Faculty of Medicine and Health Sciences, Stellenbosch University and Tygerberg Hospital, Cape Town 8000, South Africa; 3 Division of Epidemiology and Biostatistics, Department of Global Health, Faculty of Medicine and Health Sciences, Stellenbosch University, Stellenbosch 7600, South Africa; 4 Division of Epidemiology and Biostatistics, School of Public Health, Faculty of Health Sciences, Witwatersrand University, Johannesburg 2193, South Africa

**Keywords:** aortic stiffness, arterial stiffness, pulse wave velocity, HIV, HIV-associated cardiovascular disease, vascular dysfunction, early vascular ageing, vasomotor tone, cardiovascular risk

## Abstract

**Background:** People living with HIV (PLWH) are at increased risk of cardiovascular disease (CVD). Aortic stiffness measured with carotid-femoral pulse wave velocity (PWV) is a validated predictor of CVD and has been shown to be elevated in PLWH at HIV diagnosis. **Objectives:** To prospectively evaluate the effect of 9 months of contemporary antiretroviral therapy (ART) on aortic stiffness in newly diagnosed PLWH. **Methods:** A group of ART-naïve PLWH with modest cardiovascular risk and without known CVD were recruited at the time of HIV diagnosis. Aortic stiffness was measured using carotid-femoral PWV (ViCorder, Skidmore Medical, UK) before ART initiation and after 9 months on treatment. **Results:** Seventy-three participants were analysed (mean age 32 ± 7 years; 45% female). PWV decreased significantly after 9 months of ART (5.93 ± 1.0 vs. 5.69 ± 1.0 m/s; *p* = 0.01) with increased aortic distensibility (0.36 [IQR 0.30 to 0.46] vs. 0.40 [IQR 0.34 to 0.50] mmHg^−1^; *p* = 0.02). The reduction in PWV remained significant after adjustment for covariates (mean change −0.26 m/s; 95% CI −0.07 to −0.45; *p* = 0.01). **Conclusions:** Aortic stiffness improved in a group of newly diagnosed PLWH after ART initiation at 9 months, in keeping with a modifiable component of HIV-associated vascular dysfunction. The findings are consistent with reversible functional vascular abnormality, potentially mediated by changes in vasomotor tone, although it is unknown if this effect will be sustained long-term. The trajectory of aortic stiffness in PLWH and the mechanisms that underpin vasomotor tone require further study.

## 1. Introduction

The increased burden of cardiovascular disease (CVD) in people living with HIV (PLWH) is well-established. The causes, mechanisms, and how to address this increased CVD risk in these persons are unfortunately less clearly defined and are the topic of ongoing research [1].

Elevated aortic stiffness, measured using carotid-femoral pulse wave velocity (PWV), is a validated tool to predict cardiovascular events and all-cause mortality [2,3,4]. It is currently the non-invasive gold standard for aortic stiffness assessment [4,5,6] and is a composite indicator of CVD that appears to be independent of traditional risk factors [7]. PWV serves as a sensitive surrogate marker for early vascular ageing, an overarching process driven by both the irreversible structural loss of arterial elasticity (arteriosclerosis), the development of atherosclerotic plaque (atherosclerosis), and functional changes in the vessel wall [6,8,9]. In recent decades, our understanding of the arterial system with all its complexities has increased, with the propagative model being the most accepted haemodynamic framework to explain and study arterial stiffness [6,10]. Current understanding suggests that a change in PWV is either due to structural stiffening of the arterial wall (“anatomical stiffness”) or alterations in the vasomotor tone (“functional stiffness”) [8,9]. The latter is in turn dependent on endothelial function, the sympathetic nervous system and the renin–angiotensin–aldosterone system (RAAS) [11,12,13].

A recent comprehensive meta-analysis has largely addressed initial controversy on the PWV in PLWH, with current evidence in support of increased PWV in PLWH compared to their uninfected peers [7]. In addition, there is mounting evidence to suggest that this difference is already detectable at the time of HIV diagnosis, before the initiation of antiretroviral treatment (ART) [7,8,14], although the underlying mechanisms remain incompletely understood. Structural changes in the vessel wall are frequently described as the predominant reason for this [4,15], but other potentially modifiable factors that underpin vascular tone likely have a role to play as well [16]. Current data suggest an association of ART with increased PWV in the long term [7] that may be ascribed to arterio- and atherosclerosis; unfortunately, our understanding of the short- to medium-term effects of contemporary ART on PWV is suboptimal [8], and research in this setting may shed light on potentially modifiable mechanisms contributing to CVD in this population.

A particular challenge of HIV-associated CVD is the heterogeneous clinical picture of disease. HIV can affect any segment of the cardiovascular system, and the profile of disease differs between high-income countries where coronary artery disease is a major concern vs. low- to middle-income countries where myocardial disease is the leading pathology [1,17,18]. The bulk of research on risk stratification emanates from high-income countries, and our current risk prediction systems appear to be inaccurate in PLWH [19]. Data to create tailored risk stratification systems in PLWH are required, and the role of PWV appears promising as an independent risk predictor. The use of risk stratification tools in this setting, as well as their underlying pathophysiological mechanisms, require study.

We hypothesise that aortic stiffness in PLWH, as measured with PWV, will decrease after the initiation of ART due to functional, rather than established anatomical abnormalities of the vessel wall. We set out to evaluate the short-term effects of contemporary ART on PWV in a group of newly diagnosed PLWH.

## 2. Methods and Materials

### 2.1. Study Design and Participants

The study participants were recruited from the Western Cape Province of South Africa from 2020 to 2022 and consisted of newly diagnosed, HIV-infected persons, free from known CVD that has been described previously [20]. Briefly, the inclusion criteria for the study were 18 to 55 years, no history of CVD (excluding controlled hypertension), not pregnant, no acute intercurrent illness (including coronavirus disease 2019) and no known history of acquired or congenital CVD. The participants were recruited consecutively as outpatients and represent relatively healthy patients with a new diagnosis of HIV in the contemporary ART-era South Africa. After recruitment, they were started on ART according to local guidelines. Ethical approval was granted by the Stellenbosch University Human Research Ethics Committee, and written informed consent was obtained from all participants.

### 2.2. Clinical Data Collection

Comprehensive, prospective clinical data were collected as outlined elsewhere [20]. In short, data were acquired at a baseline visit before the initiation of ART and a final visit at 9 months on ART. Participants underwent morning assessments following a minimum 10 h fast and were instructed to withhold any caffeine and tobacco products on the day of the research visits. Comprehensive consultations and physical examinations were performed by the same investigator on all participants. Smoking and alcohol consumption were quantified based on provided history [8]. Manual blood pressure was measured on the right arm in a seated position after a 10 min rest, using a consistent wall-mounted sphygmomanometer unit (Welch Allyn, Skaneateles Falls, NY, USA) and a standard adult-size cuff. Larger participants were fitted with a large adult cuff covering at least two-thirds of the upper arm. Fasting blood samples were sent to the on-site National Health Laboratory Service for biochemical analysis that included kidney function, immunological and virological parameters.

### 2.3. PWV

We have previously outlined our methodology, using 10 min of averaged PWV data rather than snapshot measurements, where continuous measurements are monitored in real time to ensure high-quality, artefact-free datasets with low measurement variability [8]. PWV was measured in a temperature-controlled room using the Vicorder module (software version 8.3.7698.18341; Skidmore Medical, Bristol, UK). Aortic stiffness was expressed in terms of PWV and aortic distensibility. PWV was calculated as [direct path length (L)/transit time (TT) × 0.8]. Aortic distensibility (D) was calculated using the Bramwell-Hill equation, where D = 3.57/PWV^2^ [6].

Participants rested supine for at least 10 min prior to PWV measurement. A 100 mm blood pressure cuff was placed on the upper thigh and a 30 mm partial cuff on the neck at carotid artery level. Direct path length (L) was measured from the suprasternal angle to the top of the thigh cuff. Both cuffs were simultaneously inflated to 60 mm Hg to record the femoral and carotid waveforms. Recordings were made for at least 10 min, with automated software analysing the data and generating a PWV datapoint every 3 s. Data were validated in real-time, and individual datasets with a coefficient of variation (CV) of <30% were included for analysis. PWV measurements were standardised using a scaling factor of 0.8 [15].

### 2.4. Statistical Analysis

Statistical analyses were performed using SPSS version 29 (IBM Corporation, Armonk, NY, USA). Continuous data are presented as mean ± standard deviation. For highly skewed data, the median and interquartile ranges are reported. Categorical data are presented as frequencies with percentages. The normality of data was tested using the Shapiro–Wilk test. Significant differences between the HIV-infected group at baseline and 9 months after the initiation of ART were determined using the paired samples *t*-test or McNemar test, as appropriate. To adjust for potential confounding factors of the outcome variable PWV, a linear mixed-effects model was utilised. The model accounted for subject-specific variation in the PWV values and assumed an unstructured covariance structure. Statistical significance was defined as *p* value of <0.05.

## 3. Results

### 3.1. Study Population

73 persons completed at least 9 months of prospective follow-up after the initiation of ART and were included in the study. The median follow-up time to repeat PWV assessment was 9 months (IQR 9–13 months). Baseline characteristics of the cohort are shown in Table 1. The cohort was relatively young, with a mean age of 32 years, with good female representation (45%). Essentially all participants were on a dolutegravir-based regimen (97%), consumed less alcohol after starting therapy and gained weight after 9 months on ART. No significant change in systolic blood pressure was observed over time, with a nominal decrease in diastolic blood pressure and resting heart rate. No significant intercurrent illness was observed, with only one participant developing pulmonary tuberculosis and one mild case of proven COVID-19. After recruitment, two participants were started on antihypertensive therapy, one participant was initiated on statin treatment, and 43 participants received appropriate isoniazid preventive therapy through their local clinics in accordance with national treatment guidelines.

**Figure 1:**
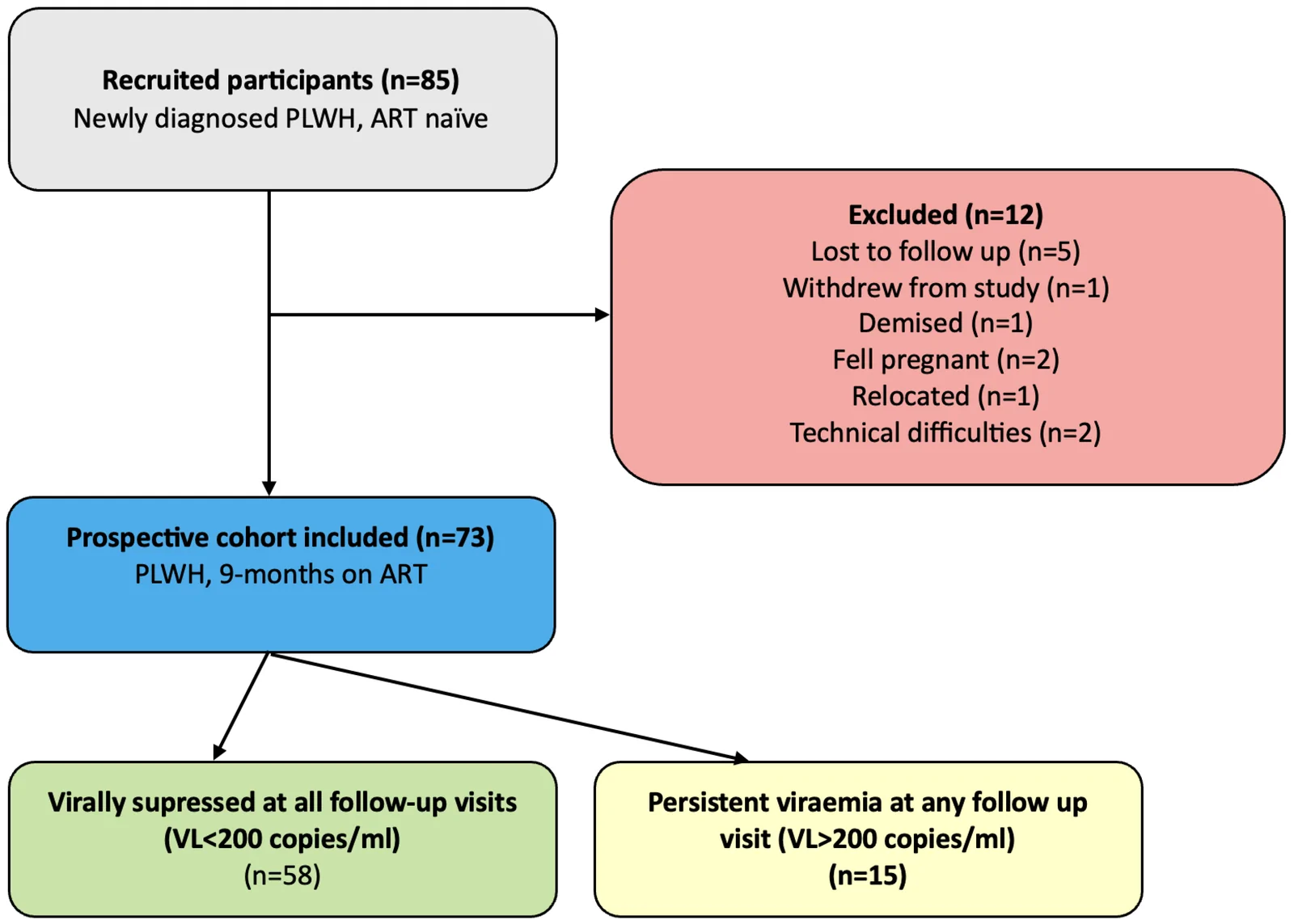
Study flow chart. PLWH = people living with HIV; ART = antiretroviral therapy; VL = HIV viral load.

Twelve participants did not complete follow-up. The reasons for not completing follow-up were unrelated to illness and were mostly due to breakdown of personal communication systems, pregnancy and non-PWV-related technical difficulties (see Figure 1).

### 3.2. Biochemical and Immunological Characteristics

Biochemical and immunological data are shown in Table 2. A significant increase in CD4 count and decrease in HIV viral load was seen 9 months after ART initiation (*p* < 0.001). At this time point, the median viral load was 20 copies/mL (IQR: 20 to 36 copies/mL), with 79% of participants achieving viral suppression (viral load < 200 copies/mL). High-sensitivity C-reactive protein (hs-CRP) decreased by 44%, while the group’s serum creatinine demonstrated an increase of 17% on combination tenofovir/lamivudine/dolutegravir.

### 3.3. Aortic Stiffness: Univariate Analysis

PWV measurements demonstrated excellent reproducibility with a median coefficient of variation of 3.3% (IQR 2.6 to 4.9%) before ART initiation and 3.1% (IQR 2.4 to 4.2%) after 9 months of ART (Table 3). Following at least 9 months of ART, the mean PWV (with 0.8 scaling factor) decreased significantly by 0.26 m/s (95% CI 0.07 to 0.45 m/s; *p* = 0.01). Median aortic distensibility demonstrated a significant difference between the groups as well, measuring 0.36 (IQR: 0.3 to 0.46 mmHg^−1^) in the ART-naïve group compared to 0.40 (IQR 0.34 to 0.50 mmHg^−1^) at 9 months on ART.

### 3.4. Aortic Stiffness: Multivariable Analysis

After adjusting for covariates (Table 4), the reduction in PWV remained significant 9 months after ART initiation (β = −0.26, 95% CI −0.45; −0.07, *p* = 0.01). This result was consistent in both the paired *t*-test analysis and in the linear mixed effects model adjusting for standard baseline covariates. Sensitivity analyses (see Supplementary Materials) excluding participants on antihypertensives, statins and salbutamol confirmed a similar, statistically significant reduction in PWV after 9 months of ART (β = −0.17, 95% CI −0.32; −0.01, *p* = 0.04). Covariates that demonstrated a statistically significant influence on PWV included age and mean arterial pressure.

## 4. Discussion

In our prospective study, we observed favourable short-term changes in aortic stiffness in a group of South African PLWH as they transitioned from an untreated state to 9 months of ART. Using PWV, we demonstrated a significant reduction in aortic stiffness 9 months after ART initiation, suggesting that at least part of the vascular dysfunction associated with early HIV infection is reversible. Interpretation of cardiovascular findings in HIV research is frequently complicated by multiple patient, viral and environmental factors, including traditional cardiovascular risk factors, established CVD, prolonged HIV duration, and heterogeneous ART exposure [21]. Our cohort was largely free of cardiovascular comorbidities, and each participant effectively served as their own control due to our longitudinal study design. By examining a young cohort with newly diagnosed HIV before and shortly after ART initiation, we were able to assess vascular changes in a setting where confounding factors were minimised.

### 4.1. Pressure Amplification

Peripheral arteries may modulate the central arterial pressure wave by means of pressure amplification, a process that is particularly evident in younger individuals [10]. Pressure amplification represents the progressive increase in pulse amplitude as the systolic pulse wave moves from the elastic, proximal aorta to the distal, stiffer peripheral arteries, resulting in a higher net amplitude of the pressure wave peripherally compared to centrally. This phenomenon underlies the recognised limitation of utilising brachial pulse pressure as a surrogate for central aortic or carotid pulse pressures [10]. However, it provides a useful theoretical framework to interpret our findings.

### 4.2. Functional Improvement in Aortic Stiffness

**Figure 2:**
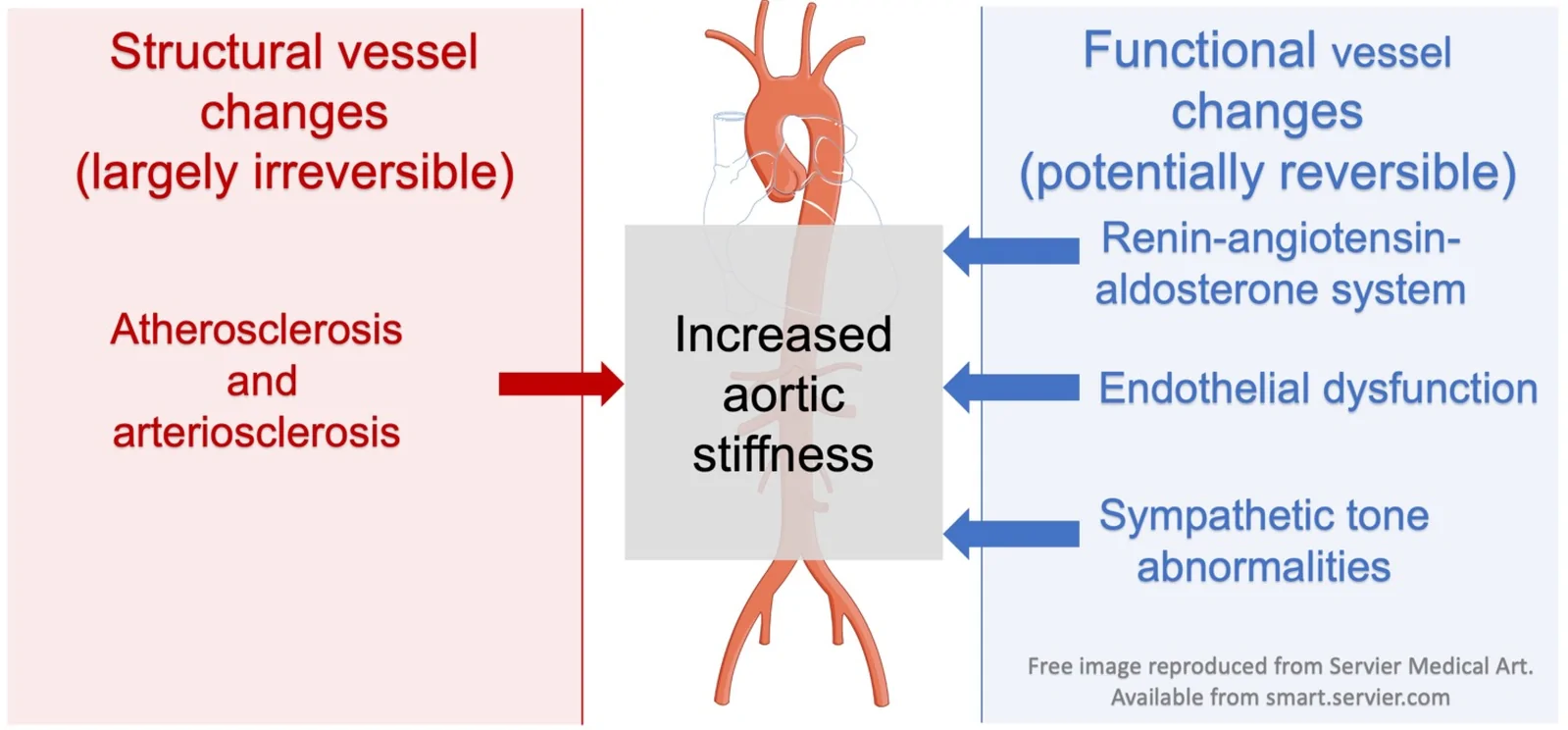
Factors influencing aortic stiffness (reproduced from [8] under a Creative Commons CC BY licence).

Conceptually, the factors affecting aortic stiffness can be broadly thought of as either (1) structural- or (2) functional vessel changes [8] (See Figure 2). The former (arteriosclerosis and atherosclerosis) represents changes that typically evolve over years and are unlikely to regress substantially over short periods [22,23]. Functional vessel changes, in contrast, are potentially reversible and are modulated by vasomotor tone that is, in turn, influenced by endothelial function, the RAAS, and sympathetic tone [6]. As these factors predominantly influence peripheral arteries, they may contribute to changes in measured PWV through their effects on pressure amplification. The short-term decrease in PWV aligns with the theoretical framework that it is more likely that vasomotor tone decreased, rather than the reversal of established arteriosclerosis and atherosclerosis. A recent randomised controlled trial evaluating the influence of statin therapy on PWV in PLWH did not demonstrate a significant difference compared to placebo, suggesting that short-term changes in PWV are unlikely to reflect modification of atherosclerosis [24].

### 4.3. Short- and Long-Term Effects of ART on Aortic Stiffness

Our group has previously demonstrated a relative increase in aortic stiffness using PWV in PLWH at the time of diagnosis compared to matched, HIV uninfected controls [8], a finding that is supported by meta-analysis data [7] as well as groups utilising cardiovascular magnetic resonance imaging and applanation tonometry [25,26]. Despite a growing interest in the use of PWV to evaluate cardiovascular health, prospective studies on the direct influence of ART on PWV remain limited. Our findings are in accordance with a small study from Mexico [27] and a larger study from Malawi [28], where a reduction in PWV was documented at 12 and 11 months, respectively. Comparison with these studies suggests that the favourable effects of ART on vasomotor tone may be seen in different population groups and across the spectrum of immunosuppression.

The long-term effects of ART on aortic stiffness remain unclear; however, cross-sectional research on treated individuals provides a glimpse of what the future may hold. Interestingly, although PWV remained lower than baseline, research from Malawi demonstrates a late increase from the nadir towards the end of follow-up, potentially a harbinger of an upward trajectory, in keeping with transient reduction in PWV after ART [28].

Meta-analysis data suggest that aortic stiffness is higher in PLWH who have been on ART for several years compared to those not on ART [7], with treated HIV being independently associated with increased aortic stiffness after years on ART, with similar magnitudes compared to metabolic syndrome [26]. Recent longitudinal research from sub-Saharan Africa showed that ART-naïve individuals have the largest increase in PWV after initiating ART at 3 years, compared to PLWH already on ART [25]. When interpreting our prospective data on this background, we observed a short-term functional decrease in PWV that may not be sustained long-term. The current body of evidence, although limited, suggests a dynamic, rather than linear relationship between HIV, ART and aortic stiffness. Untreated HIV appears to be associated with increased aortic stiffness at diagnosis, followed by a short-term improvement after ART initiation, potentially reflecting reversible functional vascular changes. Over longer periods, progressive structural vascular remodelling, ageing and cumulative cardiovascular risk may become dominant again, resulting in increasing aortic stiffness despite ART. This conceptual framework may help reconcile some of the discordant findings reported across studies performed at different stages of HIV infection and ART exposure.

### 4.4. Possible Mechanisms

While our study was not specifically designed to determine the relative contribution of individual functional factors that influence vasomotor tone, supportive findings that emanate from our work include the significant reduction in hs-CRP that evidences decreased systemic inflammation after the initiation of ART. This, together with the increased CD4 counts and decreased viral load, could contribute to functional improvements in PWV through improved endothelial function. Furthermore, the cohort’s heart rate decreased modestly, but significantly after 9 months, which may be a signal for a decrease in sympathetic tone. A better understanding of these mechanisms is required to facilitate the development of strategies to reduce cardiovascular risk in PLWH, including the identification of high-risk groups, optimisation of ART regimens, and targeted pharmacological interventions. Notably, the ART landscape has changed substantially over the past two decades, and contemporary first-line regimens are generally associated with more favourable cardiovascular risk profiles than earlier therapies. As a result, findings from historical cohorts may not fully reflect current pathophysiological patterns and mechanistic studies in the modern ART era remain limited.

### 4.5. Limitations and Strengths

The decrease in the PWV after 9 months is statistically significant; however, the clinical significance is arguably nominal, as meta-analysis data without the use of a scaling factor have estimated that for every 1 m/s increase in PWV, cardiovascular risk increases by approximately 14% [4]. However, from a mechanistic standpoint, our study provides valuable insights into the factors that stand at the theoretical core of PWV according to the propagative haemodynamic model, in turn, better informing future research approaches and avenues. Although our study has a relatively small sample size, this limitation is balanced by the homogeneity of the cohort with few confounding factors that usually plague HIV research, the longitudinal design where participants served as self-controls, and our 10 min averaged PWV methodology that provided robust, statistically significant data. The attrition rate of our cohort was relatively low (<10%), and although this could introduce bias into the study, available information on the participants who were excluded from the final analysis is predominantly random, non-disease-related and unlikely to significantly influence the results. Our study design did not include longitudinal follow-up of an HIV uninfected control group, limiting our ability to attribute the observed changes in PWV exclusively to ART. However, the prospective design in which each participant served as their own control minimised confounding from individual participant characteristics and allows for robust assessment of vascular changes during the transition from untreated HIV infection to early ART. Our findings are most directly applicable to young adults with newly diagnosed HIV initiating contemporary dolutegravir-based ART, and should not necessarily be extrapolated to older populations, individuals with established cardiovascular disease, or persons receiving alternative ART regimens. Notably, HIV is a lifelong disease, and PLWH will continue to age while on ART, accumulating both HIV-related and traditional cardiovascular risk factors, making the early vascular changes observed in this study relevant to long-term cardiovascular health, highlighting the importance of identifying potentially modifiable mechanisms underlying excess cardiovascular risk.

## 5. Conclusions

We observed modest but significant short-term improvement of aortic stiffness in a group of PLWH after 9 months of ART. The short timeframe and accompanying physiological changes suggest reversible functional rather than anatomical vascular mechanisms, although the specific pathways remain uncertain. In addition, it is unknown if this effect will be sustained long-term. Future prospective research on the trajectory of aortic stiffness in the modern ART era, as well as mechanistic research evaluating factors that underpin vasomotor tone, is required.

## Figures and Tables

**Table 1:** Study population.

Parameter	HIV Infected ART Naïve (n = 73) (Naïve)	HIV Infected 9 Months on ART (n = 73) (ART)	*p* Value ^a^
Age (years)	32 ± 7	-	-
Female sex	33 (45%)	-	-
Smoking history	36 (49%)	37 (51%)	-
Ethanol (units per week)	4 (0 to 12)	0 (0 to 11)	**0.01**
Waist circumference (cm)	80 ± 9	82 ± 10	0.06
Body mass index (kg/m^2^)	23 ± 4	24 ± 4	**<0.001**
Blood pressure (mmHg)			
Systolic	112 ± 15	112 ± 15	0.69
Diastolic	71 ± 10	68 ± 10	**0.02**
Mean arterial pressure	85 ± 11	83 ± 11	0.12
Resting heart rate on ECG (bpm)	74 ± 15	65 ± 13	**<0.001**
World Health Organisation HIV clinical stage			
I	28 (38%)	28 (38%)	-
II	19 (26%)	19 (26%)	
III	25 (34%)	24 (34%)	
IV	1 (1%)	2 (1%)	
On treatment for TB	10 (14%)	2 * (3%)	-
Pulmonary	8	0	
Extra-pulmonary	2	2	
Days since HIV diagnosis	8 (5 to 26)	300 (283 to 389)	-
Time to 9 months follow-up (months)	-	9 (9 to 13)	-
History of proven COVID-19 (mild disease)	None	1 (1%)	-
Medications			-
Salbutamol metered dose inhaler	1 (1%)	1 (1%)	
Rifampicin/isoniazid/pyrazinamide/ethambutol	10 (14%)	2 (3%)	
Trimethoprim/sulphametoxazole	21 (29%)	14 (19%)	
Isoniazid prophylaxis	1 (1%)	43 (59%)	
Pyridoxine	10 (14%)	43 (59%)	
Tenofovir/lamivudine/dolutegravir	Naïve	71 (97%)	
Losartan	-	2 (3%)	
Amlodipine/hydrochlorothiazide	1 (2%)	3 (4%)	
Statins	-	1 (1%)	
6 min walk test distance (m)	618 ± 92	621 ± 75	0.7

Bold values indicate statistical significance. Continuous variables are mean ± standard deviation or median (interquartile range) unless otherwise specified. ^a^ Naïve vs. ART; HIV = Human immunodeficiency virus; TB = Tuberculous disease; COVID-19 = Coronavirus disease-19; ART = Antiretroviral therapy; * One additional patient developed tuberculosis after recruitment.

**Table 2:** Biochemical and immunological evaluation.

Parameter	HIV Infected ART Naïve (n = 73) (Naïve)	HIV Infected 9 Months on ART (n = 73) (ART)	*p* Value ^a^
Serum creatinine (μmol/L)	70 ± 14	82 ± 16	**<0.001**
eGFR (mL/min/1.73 m^2^)	92 ± 22	83 ± 19	**<0.001**
hs-CRP (mg/L)	3.4 (0.8 to 13.7)	1.9 (0.5 to 7.5)	**0.01**
CD4 count (cells/μl)	289 (170 to 410)	378 (271 to 593)	**<0.001**
CD8 count (cells/μl)	817 (584 to 1147)	624 (446 to 858)	**<0.001**
CD4:CD8 ratio	0.35 (0.19 to 0.45)	0.60 (0.39 to 0.94)	**<0.001**
HIV viral load (copies/mL)	78,279 (9796 to 336,900)	20 * (20 to 36)	**<0.001**
HIV viral load (log copies/mL)	4.9 (4.0 to 5.5)	1.3 * (1.3 to 1.6)	**<0.001**

Bold values indicate statistical significance. Continuous data is presented as either mean ± standard deviation or median (1st quartile to 3rd quartile) unless otherwise specified. ^a^ ART naïve vs. ART group; eGFR = estimated glomerular filtration rate; hs-CRP = high sensitivity C-reactive protein; * 20 copies/mL (log = 1.3) is the lower limit of measurement for our HIV-1 assay.

**Table 3:** Carotid-femoral pulse wave velocity data.

Parameter	ART Naïve n = 73	9 Months ART n = 73	Mean Difference	95% CI of the Difference	*p*-Value
Unadjusted PWV (m/s)	7.41 ± 1.2	7.09 ± 1.1	0.33	0.08 to 0.57	**0.01**
PWV with 0.8 scaling factor (m/s)	5.93 ± 1.0	5.69 ± 1.0	0.26	0.06 to 0.46	**0.01**
Coefficient of variation (%)	3.3 (2.6 to 4.9)	3.1 (2.4 to 4.2)	-	-	0.08
Aortic distensibility (mmHg^−1^)	0.36 (0.30 to 0.46)	0.40 (0.34 to 0.50)	-	-	0.02 *

Bold values indicate statistical significance. Continuous variables are mean ± standard deviation or median (interquartile range) unless otherwise specified. *p*-values in bold indicate statistical significance using a paired *t*-test. * Log10 transformed data; CI = Confidence interval; PWV = carotid-femoral pulse wave velocity.

**Table 4:** Linear mixed effects model: Carotid-femoral pulse wave velocity as outcome variable.

Covariate	β (95% Confidence Interval)	*p*-Value
Intercept	1.69 (−0.00 to 3.38)	0.05
Male sex	0.28 (−0.08 to 0.63)	0.13
**9 months ART**	**−0.26 (−0.07 to −0.45)**	**0.01**
**Age (years)**	**0.03 (0.01 to 0.05)**	**0.01**
Smoking history	0.11 (−0.24 to 0.45)	0.55
**Mean Arterial Pressure (mmHg)**	**0.03 (0.02 to 0.05)**	**<0.001**
Resting heart rate (bpm)	0.01 (−0.00 to 0.02)	0.20

Bold values indicate statistical significance.

## Data Availability

The data presented in this study are available on request from the corresponding author and with permission from Stellenbosch University upon reasonable request due to Institutional policy—privacy restrictions.

## Supplementary Materials

The following supporting information can be downloaded at: https://www.mdpi.com/article/10.3390/diagnostics16172868/s1.
