# Supplementary material for: Improvement in Aortic Stiffness After 9 Months of Antiretroviral Therapy in Treatment-Naïve People Living with HIV
Source: Diagnostics (Basel). 2026 Sep 7;16(17):2868. doi: 10.3390/diagnostics16172868 (PMC13564491; doi:10.3390/diagnostics16172868)
Supplement: Supplementary file 1 [file diagnostics-16-02868-s001.zip › diagnostics-4404853-supplementary.pdf]

<u>Additional analyses:</u>

Linear mixed effects models with carotid-femoral pulse wave velocity as outcome variable.

Full cohort (n=73)

**Covariate**	**β (95% confidence interval)**	**p-value**
Intercept	1.84 (0.03 to 3.65)	0.05
Male sex	0.28 (-0.11 to 0.67)	0.15
**9 months ART treatment**	**-0.26 (-0.46 to -0.06)**	**0.01**
Age (years)	0.03 (0.002 to 0.05)	**0.03**
Smoking history	0.09 (-0.29 to 0.46)	0.64
Systolic Blood Pressure (mmHg)	0.02 (0.01 to 0.03)	**<0.001**
Resting heart rate (bpm)	0.01 (-0.01 to 0.02)	0.26

Bold values indicate statistical significance.

After removing 6 individuals on potentially vasoactive medications (n=67):

**Covariate**	**β (95% confidence interval)**	**p-value**
Intercept	2.13 (0.39 to 3.87)	0.02
Male sex	0.35 (-0.03 to 0.72)	0.07
**9 months ART treatment**	**-0.17 (-0.32 to -0.01)**	**0.04**
Age (years)	0.02 (-0.00 to 0.05)	0.08
Smoking history	0.13 (-0.23 to 0.48)	0.48
**Systolic Blood Pressure (mmHg)**	**0.02 (0.01 to 0.03)**	**<0.001**
Resting heart rate (bpm)	0.01 (-0.00 to 0.02)	0.22

Bold values indicate statistical significance.

Replaced the covariate systolic blood pressure with mean arterial pressure:

Full cohort (n=73)

**Covariate**	**β (95% confidence interval)**	**p-value**
Intercept	1.69 (-0.00 to 3.38)	0.05
Male sex	0.28 (-0.08 to 0.63)	0.13
**9 months ART treatment**	**-0.26 (-0.45 to -0.07)**	**0.01**
**Age (years)**	**0.03 (0.01 to 0.05)**	**0.01**
Smoking history	0.11 (-0.24 to 0.45)	0.55
**Mean Arterial Pressure (mmHg)**	**0.03 (0.02 to 0.05)**	**<0.001**
Resting heart rate (bpm)	0.01 (-0.00 to 0.02)	0.20

Bold values indicate statistical significance.

After removing 6 individuals on potentially vasoactive medications (n=67):

**Covariate**	**β (95% confidence interval)**	**p-value**
Intercept	1.75 (-0.03 to 3.54)	0.05
Male sex	0.33 (-0.03 to 0.70)	0.08
**9 months ART treatment**	**-0.17 (-0.32 to -0.01)**	**0.04**
**Age (years)**	**0.02 (0.00 to 0.05)**	**0.04**
Smoking history	0.13 (-0.22 to 0.48)	0.47
**Mean Arterial Pressure (mmHg)**	**0.03 (0.02 to 0.05)**	**<0.001**
Resting heart rate (bpm)	0.01 (-0.00 to 0.02)	0.19

Bold values indicate statistical significance.
